# Study on the Mechanism of Eco-Friendly Hydrogel in Enhancing Condensation Water Utilization by Vegetation in Rocky Mountainous Areas

**DOI:** 10.3390/plants15121832

**Published:** 2026-06-13

**Authors:** Dan Ma, Shuai Zhang, Weijie Yuan, Yong Gao

**Affiliations:** 1 College of Desert Control Science and Engineering, Inner Mongolia Agricultural University, Hohhot 010018, China; madan_1001@163.com (D.M.); 13948815709@163.com (Y.G.); 2 Experimental Centre of Forestry in North China, Chinese Academy of Forestry, Beijing 102300, China

**Keywords:** foliar water uptake, water-use strategy, *Pinus tabuliformis*, *Platycladus orientalis*, isotope tracing

## Abstract

In rocky mountainous regions characterized by shallow, barren soils and water scarcity, non-rainfall water, such as condensation, plays a crucial ecological role in mitigating seasonal drought in forest trees. To enhance the water-use capacity of vegetation, this study utilized a previously developed eco-friendly PVA–CS/SA–Ca^2+^ hydrogel. The primary objective was to elucidate the synergistic mechanisms by which the hydrogel optimizes condensed water utilization and drives the ecophysiological recovery of *Pinus tabuliformis* and *Platycladus orientalis*, two keystone afforestation species in northern China. Utilizing a controlled environmental chamber to simulate the condensation and humidification process, the experiment established three treatments: a control group (CK), a pot-sealed group (PS, to isolate soil water absorption), and a hydrogel-amended group (Hydrogel-Root Wrapping, HRW). To comprehensively evaluate the water utilization mechanisms, the amount of condensed water captured by the system was quantified, and hydrogen isotope tracing techniques were employed to precisely track water transport pathways and contribution rates. Concurrently, key physiological parameters were systematically determined, including leaf water potential, stomatal conductance, leaf water content, net photosynthetic rate, and transpiration rate. The results demonstrated the following: (1) the hydrogel significantly enhanced the condensation water capture capacity of the system. The net mass gains of the *Pinus tabuliformis* and *Platycladus orientalis* systems under the HRW treatment reached 26.3 g and 32.9 g, respectively, which represented 1.17 and 1.30 times those of the CK treatment, and 1.52 and 1.54 times those of the PS treatment. (2) Isotope tracing confirmed that both tree species possess significant Foliar Water Uptake (FWU) capacity. Following condensation, the δ^2^H values in the leaves of *Platycladus orientalis* and *Pinus tabuliformis* surged to 113.5‰ and 85.3‰, respectively, with stem δ^2^H values increasing by 31‰ and 22‰ compared to their initial baseline. (3) The introduction of the hydrogel in the HRW treatment provided 11.2% and 10.9% of the stem water supply for *Platycladus orientalis* and *Pinus tabuliformis*, respectively, thereby reducing their dependence on soil water by 8.3% and 13.1%. In contrast, there was no significant difference in the fractional contribution of condensation water to stem water between the PS and CK treatments. (4) Regarding physiological responses, the application of the hydrogel material effectively improved the physiological status of the plants. The leaf water potentials of *Pinus tabuliformis* and *Platycladus orientalis* increased to −0.15 MPa and −1.32 MPa, respectively. Concurrently, stomatal conductance (3.25 and 3.64 mm·s^−1^) and leaf water content (58.4% and 67.4%) were significantly higher than those in the other treatments. In summary, the hydrogel can significantly enhance the capture, conversion, and utilization efficiency of condensation water by vegetation, effectively optimizing the water supply dynamics of the system. This provides key theoretical and technical support for ecological afforestation in difficult sites within rocky mountainous areas.

## 1. Introduction

Vegetation, as a core component of terrestrial ecosystems, plays an irreplaceable role in water conservation, the maintenance of global carbon storage, and the regulation of the climate system. However, exacerbated by global warming driven by greenhouse gas emissions, the frequency and intensity of extreme weather events (such as sustained high temperatures and multi-year droughts) are continuously increasing, exposing global ecosystems to severe vulnerability risks [1,2]. Under this backdrop of climate change, water availability has emerged as the primary limiting factor constraining vegetation growth, survival, and ecosystem productivity [3]. Shifts in precipitation regimes and the ensuing soil moisture deficit not only directly inhibit key physiological processes in plants, such as photosynthesis, transpiration, and root development [4,5], but also further exacerbate the risks of land degradation and desertification in arid and semi-arid regions globally [6]. Compounding these severe ecological threats, as future climate warming and regional droughts become increasingly prevalent, available water resources will further diminish, and the water stress faced by plants will continue to intensify. Therefore, investigating the water acquisition and utilization strategies of vegetation in arid environments has become a critical focus for responding to climate change and maintaining ecosystem stability.

Habitats in the rocky mountainous regions of northern China are typically characterized by shallow soils, high gravel content, and extremely poor surface water retention capacity, leading to low overall water utilization in the region [7,8,9]. Compounded by the extreme spatial heterogeneity and seasonal imbalance of precipitation, regional vegetation frequently encounters severe and prolonged seasonal drought stress during the dry season. When soil moisture is insufficient to sustain plant survival, non-rainfall water inputs (NRWIs), particularly condensation (e.g., dew and fog), play a crucial alternative and supplementary role in alleviating plant water deficits [10,11]. Although the absolute volume of a single condensation event is relatively small, its high frequency and stable availability during the dry season [12,13] make it an indispensable, persistent water source in these fragile habitats. Crucially, a growing body of evidence indicates that plants can not only utilize condensation that drips into the shallow soil but also directly absorb it through specialized leaf surface structures (e.g., stomata and trichomes) via foliar water uptake (FWU) [14,15]. When deep soil moisture is depleted, this direct foliar water absorption mechanism can bypass the resistance of the soil–root pathway, rapidly improving plant water status, restoring leaf water potential and turgor pressure, and thereby ensuring the normal functioning of key physiological activities such as photosynthesis [16,17]. Therefore, condensation is not merely a lifeline for plants in rocky mountainous areas to survive drought periods, but also a core driver maintaining ecosystem water balance and community stability in this region.

To maximize the ecological benefits of limited water resources in arid and semi-arid regions, polymer hydrogels, characterized by excellent hygroscopic and water retention properties, have been increasingly introduced into the fields of agriculture and ecological restoration in recent years. Extensive previous studies have established that hydrogels act as dynamic soil reservoirs, substantially improving soil moisture retention and enhancing plant drought resilience [18]. Parallel to agricultural applications, breakthrough research in atmospheric water harvesting (AWH) has engineered novel hydrogels—such as binary salt-modified alginates [19], thermoresponsive zwitterionic microgels [20], and macroporous networks [21]—that are capable of rapidly capturing atmospheric vapor across diverse humidities and efficiently releasing it as liquid water. However, while the independent capabilities of agricultural moisture buffering and AWH systems are well documented, integrating high-performance AWH hydrogels directly into the plant rhizosphere to explicitly capture and utilize condensation water has remained a critical unexplored frontier.

Addressing this gap, our research group previously developed an environmentally friendly PVA–CS/SA–Ca^2+^ hydrogel [22,23]. This material was specifically designed to undergo repeated cycles of water absorption and desorption. This cyclic property perfectly aligns with the high-frequency dynamic patterns of condensation water in rocky mountainous habitats, namely nocturnal generation and diurnal dissipation, thereby allowing for the continuous and efficient capture of system water for vegetation utilization, marking the first time this ecohydrological mechanism has been theoretically and experimentally realized in the plant rhizosphere.

However, within the complex soil–plant–atmosphere continuum (SPAC), the precise extent to which plants utilize this hydrogel-enriched condensation water, alongside its transport pathways and physiological contributions within the plant body, remains unclear. Traditional moisture determination methods struggle to differentiate the specific sources of water within plants. Stable isotope tracing technology provides a robust tool to overcome this challenge. Given that water from distinct sources possesses unique isotopic signatures, the continuous monitoring of the isotopic composition of leaf and stem water enables the precise tracking of water absorption, transport, and its allocation in photosynthetic metabolism [24,25]. Building upon this, the present study constructed an artificial microhabitat using the novel environmentally friendly hydrogel, coupled with high-abundance deuterium (δ^2^H) tracing technology. A simulated condensation experiment was conducted focusing on *Pinus tabuliformis* and *Platycladus orientalis*, two principal afforestation tree species in the rocky mountainous regions of northern China.

This study aims to (1) ascertain the enhancing effect of hydrogel application on the capture of atmospheric condensation water, as well as the absorption and retention efficiency of soil condensation water; (2) quantify the direct foliar water uptake capacity of plants during condensation events; (3) reveal the recovery process of plant physiological water status (e.g., leaf water potential and stomatal conductance) under the synergistic water replenishment of the hydrogel and condensation water. Overall, by integrating stable hydrogen isotope tracing with comprehensive physiological assessments, this study is expected to provide critical mechanistic insights into how eco-friendly hydrogels modulate foliar water uptake and optimize water supply dynamics within the soil–plant continuum. Furthermore, elucidating the synergistic interaction between artificial microhabitat modification and non-rainfall water utilization has the potential to offer a robust theoretical foundation and a highly practical technical approach for enhancing the survival and ecological adaptability of plantations in water-scarce rocky mountainous regions.

## 2. Materials and Methods

### 2.1. Experimental Setup for Artificial Condensation Simulation

**Figure 1:**
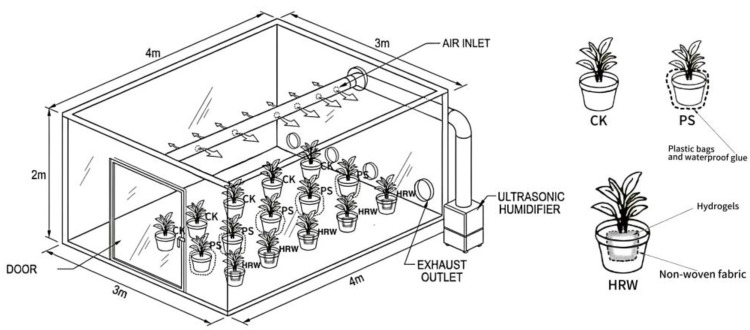
Schematic Diagram of a Controlled-environment chamber.

This experiment, spanning two consecutive clear days, was conducted in August 2025 at the National Observation and Research Station of the Capital Circle Forest Ecosystem, Beijing Forestry University, located in Jiufeng. Prior to the experiment, a custom-built controlled-environment chamber (4 m × 3 m × 2 m) was constructed. The framework of the chamber was fabricated from angle steel and tightly enclosed with a customized, detachable transparent waterproof tarpaulin. The assembled chamber was connected via PVC pipes to an ultrasonic humidifier (DRS-06A, Dorosin, Guangzhou, China) with a nebulization rate of 6 L/h and a droplet size range of 1–5 μm Air outlets were configured along the indoor PVC pipes to discharge the condensation mist. Additionally, an exhaust port was installed on the side of the chamber to vent excess mist in the event of supersaturation. Through these components, a complete artificial condensation simulation system was established (Figure 1).

### 2.2. Experimental Design

The experiment selected *Pinus tabuliformis* and *Platycladus orientalis*, the primary afforestation tree species in the rocky mountainous regions of northern China, as the experimental subjects. Three-year-old seedlings of both species were planted in pots (top diameter 33.5 cm, height 33.3 cm) filled with leached cinnamon soil. The selected seedlings exhibited uniform growth traits, including height, diameter at breast height (DBH), and crown spread (Table 1). To control the baseline range of isotopic abundance in plant tissues, all plants were subjected to consistent pre-treatment conditions and irrigated with groundwater prior to the experiment.

For this experiment, the two target plant species were each assigned to three treatment groups, with 5 replicates per group. The treatments were designated as follows: Control treatment (CK): Received no intervention. Pot-sealed treatment (PS): The base of the plant stem was hermetically sealed using plastic bags and waterproof sealant to isolate the soil from atmospheric water vapor and ensure no water leakage within the system. Hydrogel addition treatment (Hydrogel-Root Wrapping, HRW): The eco-friendly hydrogel used in this study is primarily synthesized from polyvinyl alcohol (PVA), chitosan (CS), sodium alginate (SA), and calcium ions (Ca^2+^) via chemical cross-linking and chelation [22,23]. Microscopically, this hydrogel exhibits a distinct core–shell structure: the core consists of a loose, highly developed porous three-dimensional network, while the outer shell is relatively dense. This unique spatial configuration endows the material with robust hydrophilicity and exceptional water retention capacity, achieving a maximum swelling ratio of 50 g/g. Prior to application on the experimental plants, the dry hydrogel required full hydration. Specifically, 20 g of dry hydrogel granules were continuously soaked in deionized water until reaching a swelling equilibrium state (absorbing 1000 g of water, equivalent to 100% of its maximum water absorption capacity). For each potted plant in the HRW treatment, the root system and root ball within the 0–20 cm soil layer were wrapped in non-woven gauze evenly coated with the hydrated hydrogel material, ensuring that the upper edge of the wrapped hydrogel was flush with the soil surface.

The experiment adopted a saturated humidification approach, with the humidifier set to 100% humidity, utilizing isotopically labeled condensation water prepared with deuterium-enriched water (δ^2^H ≈ 500‰) for the humidification process. The experiment was conducted over two consecutive clear days. In the study area, the nocturnal relative air humidity during summer is notably high; as both surface temperature and dew point temperature decrease at night, atmospheric moisture begins to condense. Based on the relative humidity and condensation generation patterns within the study area, the period exhibiting the maximum condensation volume (22:00 h to 06:00 h the following day) was selected as the humidification and condensation duration, lasting for a total of 8 h. To prevent supersaturation of indoor humidity, which would result in excessive condensation water adhering to the leaf surfaces, the exhaust port was opened when the relative humidity reached 100% to maintain a stable indoor relative humidity and avoid supersaturation.

Following the initiation of humidification, the entire experimental plant system was weighed hourly using a precision balance with a readability of 0.1 g (ME104E, METTLER TOLEDO, Greifensee, Switzerland), totaling 8 measurements. Upon completion of the experiment, the transparent waterproof tarpaulin of the artificial condensation simulation setup was completely removed to allow for gas exchange between the plants and the external ambient environment.

### 2.3. Measurement of Plant Physiological Parameters

Leaf water potential (Ψleaf, MPa) was measured using a dew point potentiometer (Psypro, Wescor, Logan, UT, USA). Measurements were conducted at midday (12:00 h) both prior to and following the artificial condensation simulation experiment for the CK, PS, and HRW treatment groups, with five replicates per group.

To investigate the effect of foliar water uptake on transpiration, stomatal conductance (Gs, mm·s^−1^) was measured using a porometer (AP4, Delta-T Devices, Cambridge, UK). For the CK, PS, and HRW treatments, the exact same leaves were selected and measured at the same time of day (12:00 h) before and after the experiment. Following the completion of the condensation simulation, the surface moisture of the leaves was thoroughly wiped dry before conducting the measurements. Five replicates were performed for each group.

To determine the impact of simulated condensation on leaf water content (LWC, %). Specifically, three mature leaves were sampled from the experimental plants in the CK, PS, and HRW groups at 12:00 h both before and after the experiment. The surface moisture of the collected leaves was gently wiped dry, after which their fresh and dry weights were determined using a precision balance to calculate the leaf water content. The formula is:

(1) $$W_{L}=\left(m_{f}-m_{d}\right)/m_{f}\times 100\%$$

where *m_f_* is the fresh weight of the leaf (g), and *m_d_* is the dry weight of the leaf (g).

To evaluate changes in plant water use strategies, a portable photosynthesis system (LI-6400, LI-COR, Lincoln, NE, USA) was used to measure the net photosynthetic rate (P_n_, µmol CO_2_ m^−2^ s^−1^) and transpiration rate (Tr, mmol H_2_O m^−2^ s^−1^) of the leaves. The measurement was conducted on mature leaves exposed to good light conditions at the same time point (12:00 h) before and after the experiment. Before the measurement, the attached water on the leaf surface had to be gently wiped off. The formula for water use efficiency (WUE, µmol CO_2_ mmol^−1^ H_2_O) is [26]:

(2) $$WUE=\frac{P_{n}}{T_{r}}$$

where *P_n_* is the net photosynthetic rate (µmol CO_2_ m^−2^ s^−1^), and *T_r_* is the transpiration rate (mmol H_2_O m^−2^ s^−1^).

### 2.4. Stable Isotope Sampling and Analysis

For stable isotope analysis, deionized water and groundwater samples were collected prior to the experiment, stored in 20 mL glass vials, and sealed with Parafilm. To accurately capture the instantaneous isotopic signatures of the absorbed condensate and prevent losses from daytime isotopic fractionation and transpiration, plant stem and leaf samples at uniform heights were collected immediately after the simulated humidification period concluded at 06:00. Five replicates were taken for each treatment. Crucially, immediately following collection, the surfaces of the plant samples were rapidly and thoroughly wiped dry to remove any attached external condensate, thereby preventing the internal tissue water from isotopic contamination. The samples were then swiftly placed into 20 mL glass vials, tightly sealed with Parafilm to prevent evaporation, and stored in a freezer at −20 °C to avoid isotopic fractionation caused by moisture loss.

In the laboratory, water from the plant tissues was extracted using an automatic cryogenic vacuum distillation system. The stable isotope ratios were then measured using a Thermo Scientific Delta V Advantage isotope ratio mass spectrometer (Thermo Fisher Scientific, Bremen, Germany). The raw abundance ratios measured by the instrument were standardized against the Vienna Standard Mean Ocean Water (VSMOW) and expressed in per mil notation (δ) as follows:

(3) $$\delta X=\left(\frac{R_{sample}}{R_{standard}}-1\right)\times 1000$$

where δX represents δ^2^H; *R_sample_* denotes the isotopic abundance ratio of the sample; and *R_standard_* represents the isotopic abundance ratio of VSMOW.

Based on the principle of isotopic mass conservation, the Iso-Source mixing model [27] was employed to calculate the fractional contributions of various water sources to the experimental plants. The δ^2^H values of three distinct water sources (soil water, condensate, and hydrogel) were simultaneously inputted into the model:

(4) $$\delta X_{s}=c_{1}\delta X_{1}+c_{2}\delta X_{2}+c_{3}\delta X_{3}$$

(5) $$c_{1}+c_{2}+c_{3}=1$$

where $\delta X_{s}$ represents the δ^2^H (‰) of the stem xylem water; $\delta X_{1}$, $\delta X_{2}$, and $\delta X_{3}$ denote the δ^2^H of the soil water, condensate, and hydrogel, respectively; and *c*_1_, *c*_2_, and *c*_3_ correspond to the fractional contributions of the soil water, condensate, and hydrogel to the plant water supply, respectively.

### 2.5. Statistical Analysis

The statistical analyses were performed using SPSS software (Version 22.0 IBM Corp., Armonk, New York City, NY, USA) Descriptive statistics were used to calculate the means and standard deviations for each set of replicates.

For the physiological variables and derived indices evaluated before and after the simulated condensation treatment, linear mixed-effects models were used to account for the repeated-measurement structure of the data. These variables included leaf water potential, stomatal conductance, leaf water content, net photosynthetic rate, transpiration rate, and water use efficiency. Among them, water use efficiency was calculated from the corresponding net photosynthetic rate and transpiration rate for each replicate. The models were fitted separately for each tree species and each variable or index. Treatment, measurement stage, and their interaction were included as fixed effects, whereas pot identity was included as a random effect, with measurement stage specified as the repeated factor within each pot.

Because the significance annotations in the figures were intended to compare treatment effects after condensation, pairwise comparisons among CK, PS, and HRW were conducted at the post-experiment stage based on estimated marginal means with Bonferroni adjustment. Different lowercase letters indicate significant differences among treatments at the post-experiment stage. Statistical significance was set at *p* < 0.05. Data visualization was performed using Origin 2024.

## 3. Results

### 3.1. Variations in Temperature and Humidity Inside and Outside the Controlled-Environment Chamber

**Figure 2:**
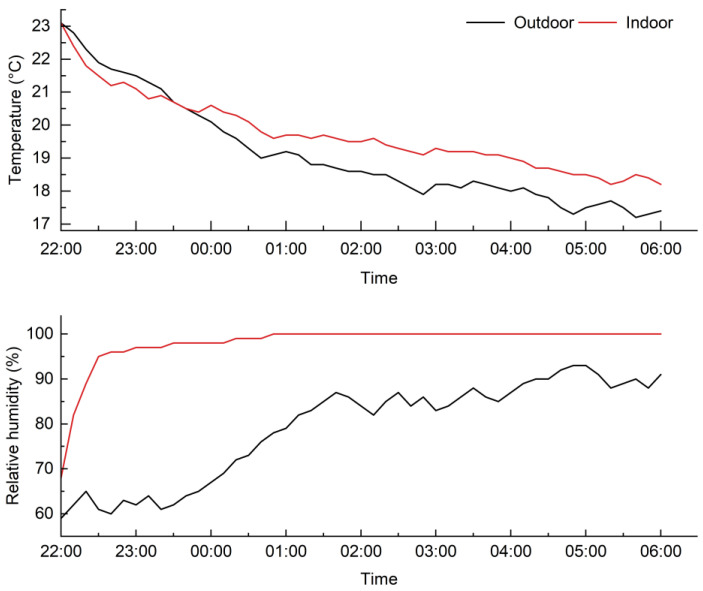
Temperature and Relative Humidity Variations Inside and Outside the Chamber.

During the experiment, portable temperature and humidity data loggers (U23-001, Onset HOBO, Bourne, MA, USA), featuring an accuracy of ±0.21 °C (at 0–50 °C) and ±2.5% RH, were suspended at plant canopy height both inside and outside the controlled-environment chamber to compare the internal and external environmental conditions. During the treatment period, temperature and humidity were logged at 10 min intervals, yielding a total of 49 recordings. The temperature difference between the inside and outside of the chamber was minimal; both gradually decreased from the onset of the experiment, reaching their minimum values at 06:00 the following day. The indoor temperature ranged from 17.5 to 23.1 °C, while the outdoor temperature varied between 18.2 and 23.1 °C. The indoor relative air humidity increased gradually at the beginning of the treatment, reached saturation at 1 h 50 min, and subsequently remained constantly at 100% until the conclusion of the experiment. The outdoor relative air humidity increased progressively from the start of the experiment, peaking at approximately 91% around 06:00. The dynamic changes in temperature and humidity inside and outside the controlled-environment chamber are illustrated in Figure 2.

### 3.2. Differences in System Water Acquisition Capacity Under Different Treatments

**Figure 3:**
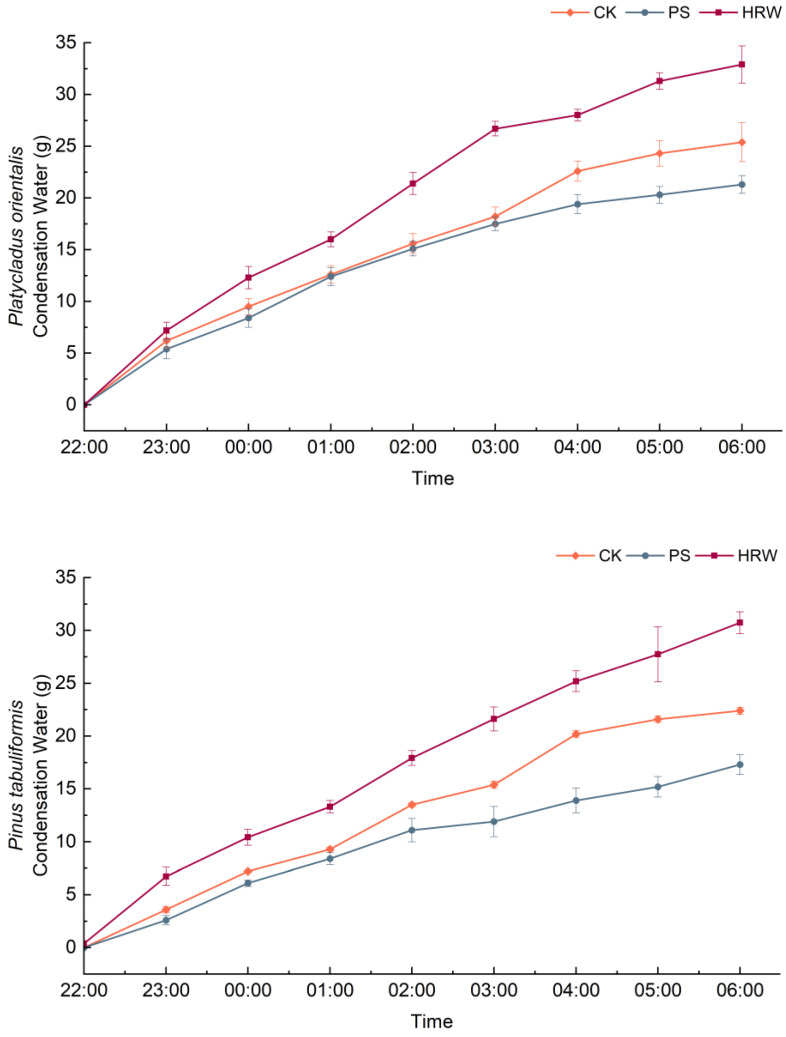
Condensate Weights of Two Tree Species.

The curves of net mass gain for the various treatment groups of *Pinus tabuliformis* and *Platycladus orientalis* during the simulated humidification period are illustrated in Figure 3. During the 8 h humidification period, the cumulative net mass gain of the whole potted plant system increased with humidification time, and the final net mass gain followed the order HRW > CK > PS. By the end of the humidification process at 06:00, the system net mass gains for *Pinus tabuliformis* under CK, PS, and HRW were 22.4 g, 17.3 g, and 26.3 g, respectively. For *Platycladus orientalis*, the corresponding values were 25.4 g, 21.3 g, and 32.9 g, respectively.

Because the entire potted plant system was weighed, the net mass gain shown in Figure 3 represents whole-system net retention of externally supplied condensation water rather than water uptake by a single component. In the PS treatment, where the pot surface was sealed, the net mass gain mainly reflected condensation retained on aboveground plant surfaces and potential foliar water uptake. Therefore, the difference between CK and PS can be regarded as an estimate of the additional contribution of the exposed soil surface to condensation retention, whereas the difference between HRW and CK indicates the additional net retention associated with hydrogel amendment relative to the soil-only system.

### 3.3. Variations in Stable Hydrogen Isotope Composition of Plant Leaves and Stems

**Figure 4:**
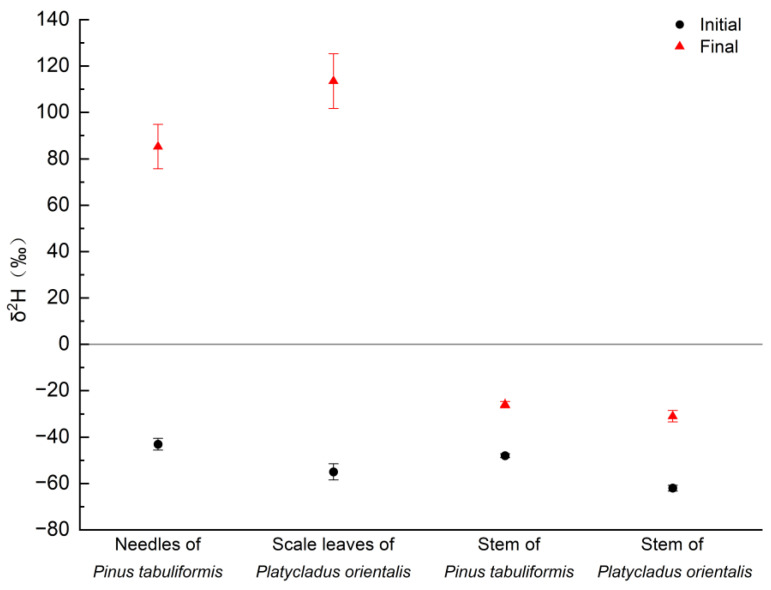
Variations in Deuterium Content of Leaves and Stems of Two Tree Species Before and After Treatment.

The isotopic labeling results (Figure 4) clearly reveal the uptake and distribution of condensation water within the plants. Following the isotope-labeled humidification experiment, the δ^2^H values in both the leaves and stems of *Pinus tabuliformis* and *Platycladus orientalis* exhibited significant shifts compared to their pre-experiment (Initial) levels. The foliar δ^2^H values of both tree species surged into the positive range, with the average value reaching approximately 113.5‰ for *Platycladus orientalis* and 85.3‰ for *Pinus tabuliformis*. Although the stem δ^2^H values of *Pinus tabuliformis* and *Platycladus orientalis* remained within the negative range, they became enriched by 22‰ and 31‰, respectively, compared to their initial baselines.

### 3.4. Sources of Stem Water Under Different Treatments

**Figure 5:**
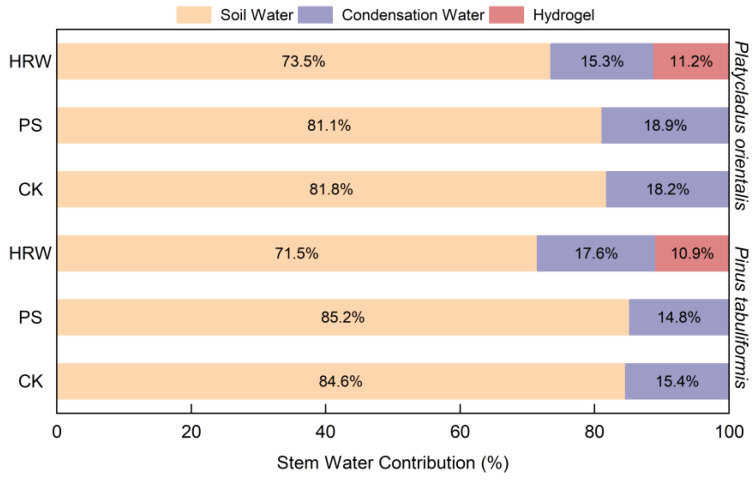
Stem Water Contribution of Two Tree Species in Treatment and Control Groups.

Through the analysis using a stable hydrogen isotope linear mixing model, the proportional contributions of different water sources to the plant stem water were quantified (Figure 5). Following the humidification experiment, the results indicated that the stem water composition of both plant species varied significantly across the different treatments.

Under the PS treatment, the fractional contributions derived from foliar water uptake in the stem water of *Platycladus orientalis* and *Pinus tabuliformis* reached 18.9% and 14.8%, respectively; whereas in the CK treatment, these proportions were 18.2% and 15.4%, respectively. The water source composition profiles of the CK and PS treatments were highly similar, consisting solely of soil water and condensation water. In the HRW treatment, the fractional contribution of water derived from the hydrogel reached 11.2% and 10.9% for *Platycladus orientalis* and *Pinus tabuliformis*, respectively.

### 3.5. Physiological Responses of Plant Leaves Following Condensation Humidification

#### 3.5.1. Variations in Leaf Water Potential After Simulated Condensation Humidification

**Figure 6:**
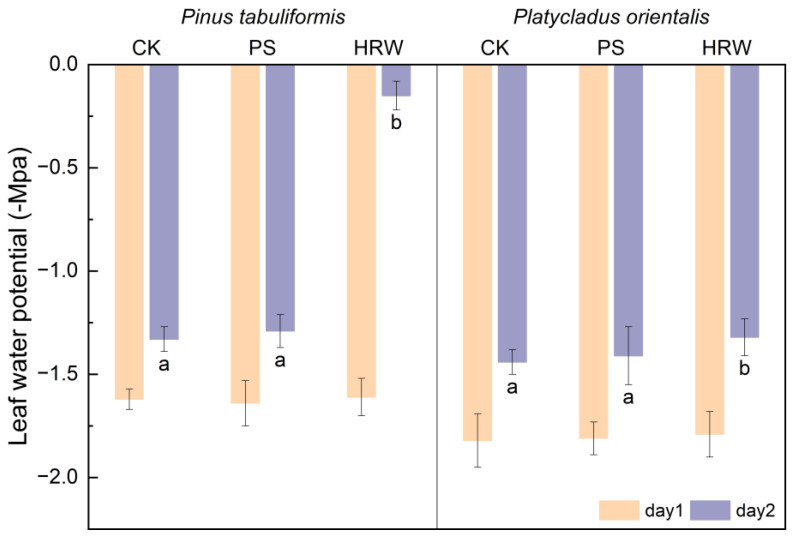
Leaf Water Potential of Two Tree Species in Treatment and Control Groups (*p* < 0.01). Different lowercase letters indicate significant differences among treatments (CK, PS, HRW) on the second day (post-experiment). No letters are shown for the pre-experiment state as there were no initial significant differences among the groups.

Leaf water potential (Ψleaf) is a direct physiological parameter for assessing the degree of plant water deficit and water acquisition capacity. Prior to the simulated humidification experiment (day 1), the midday leaf water potentials across all treatment groups of *Pinus tabuliformis* and *Platycladus orientalis* were at relatively low levels, with no significant differences observed among the groups, demonstrating the consistency of the initial experimental conditions (Figure 6). Following the humidification treatment (day 2), the leaf water potentials of all plants exhibited varying degrees of recovery. Post-experiment, the difference in leaf water potential between the CK and PS treatments was not significant, whereas the HRW treatment exhibited a significant increase. Specifically, the leaf water potential in the HRW treatment for *Pinus tabuliformis* surged to −0.15 MPa, while that for *Platycladus orientalis* recovered to −1.32 MPa.

#### 3.5.2. Variations in Stomatal Conductance After Simulated Condensation Humidification

**Figure 7:**
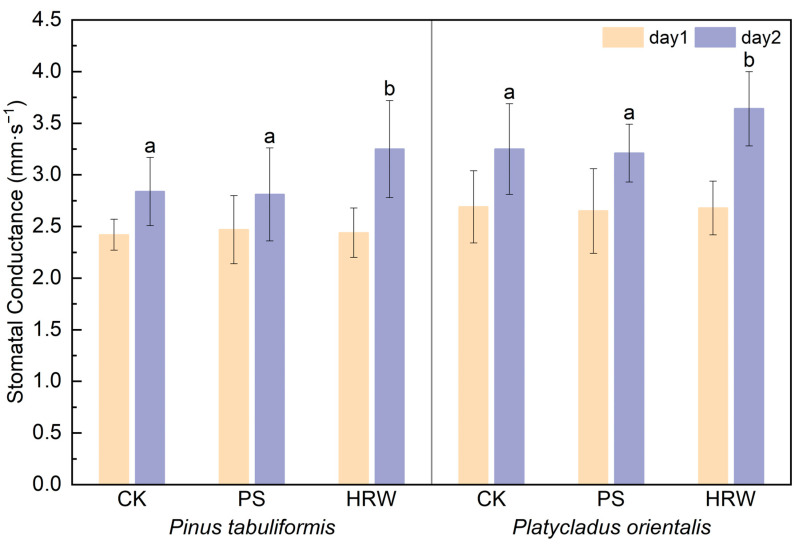
Leaf Stomatal Conductance of Two Tree Species in Treatment and Control Groups (*p* < 0.01). Different lowercase letters indicate significant differences among treatments (CK, PS, HRW) on the second day (post-experiment). No letters are shown for the pre-experiment state as there were no initial significant differences among the groups.

Stomatal conductance (Gs) is a crucial parameter for evaluating plant water utilization and the recovery of photosynthetic activity. The experimental results (Figure 7) demonstrated that prior to simulated humidification (day 1), the stomatal conductance across all treatment groups for *Pinus tabuliformis* and *Platycladus orientalis* was at relatively low levels, with no significant differences observed among the groups. Following the humidification treatment (day 2), the stomatal conductance of all plants exhibited a recovery trend. The difference in post-experiment Gs values between the CK and PS treatments was not significant. In contrast, the HRW treatment significantly increased stomatal conductance. The Gs value in the HRW treatment for *Pinus tabuliformis* increased to 3.25 mm·s^−1^, while that for *Platycladus orientalis* reached 3.64 mm·s^−1^, both of which were significantly higher than those of their corresponding CK treatments.

#### 3.5.3. Variations in Leaf Water Content After Simulated Condensation Humidification

**Figure 8:**
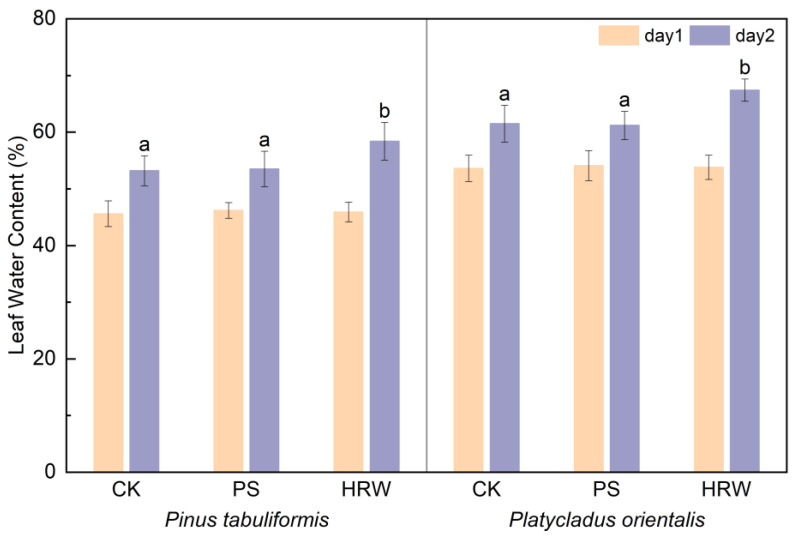
Leaf Water Content of Two Tree Species in Treatment and Control Groups (*p* < 0.01). Different lowercase letters indicate significant differences among treatments (CK, PS, HRW) on the second day (post-experiment). No letters are shown for the pre-experiment state as there were no initial significant differences among the groups.

Leaf water content (LWC) is a vital parameter reflecting the hydration status and drought tolerance of plant tissues. Prior to the simulated humidification experiment (day 1), the LWC across all treatment groups for *Pinus tabuliformis* and *Platycladus orientalis* were at relatively low levels, with no significant differences observed among the groups (Figure 8). Following the humidification treatment (day 2), the LWC of all experimental plants significantly increased. Notably, the LWC of the CK and PS treatments exhibited highly synchronous trends. The HRW treatment demonstrated the highest rehydration efficacy. By the end of the humidification process, the LWC in the HRW treatment for *Pinus tabuliformis* increased to 58.4%, while that for *Platycladus orientalis* reached 67.4%, both of which were significantly higher than those in their corresponding CK and PS treatments.

#### 3.5.4. Variations in Photosynthetic Characteristics and Water Use Efficiency of Pinus Tabuliformis and Platycladus Orientalis After Simulated Condensation Humidification

**Figure 9:**
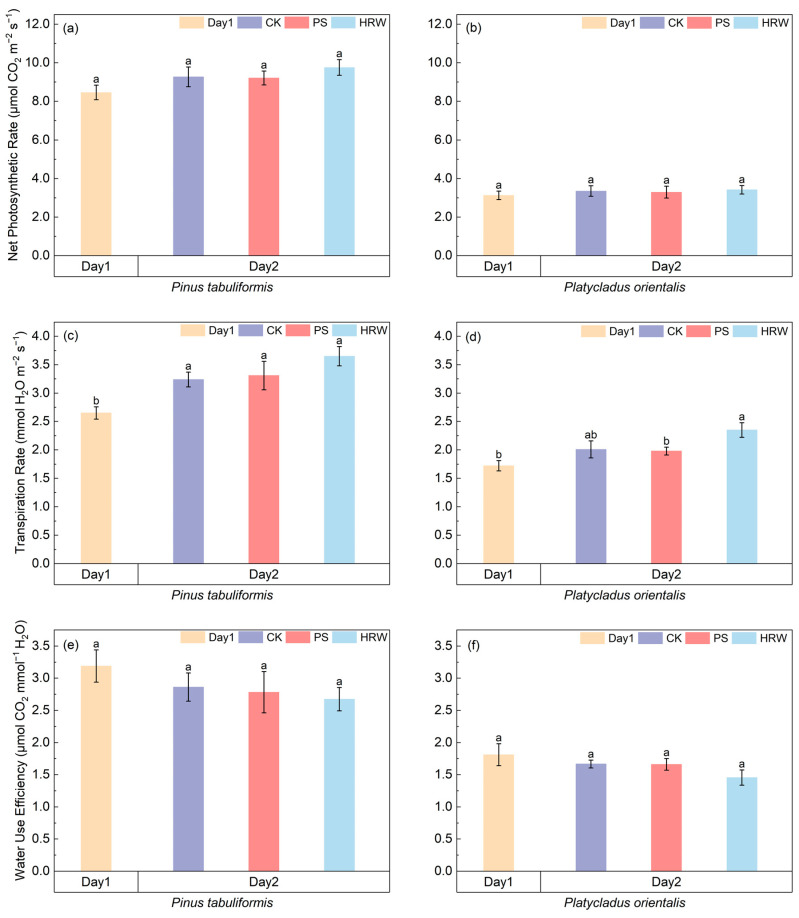
Effects of Treatment and Control Groups on Net Photosynthetic Rate, Transpiration Rate, and Water Use Efficiency of Two Tree Species. (**a**) Net photosynthetic rate of *Pinus tabuliformis*; (**b**) net photosynthetic rate of *Platycladus orientalis*; (**c**) transpiration rate of *Pinus tabuliformis*; (**d**) transpiration rate of *Platycladus orientalis*; (**e**) water use efficiency of *Pinus tabuliformis*; (**f**) water use efficiency of *Platycladus orientalis*. Different lowercase letters indicate significant differences among Day 1, CK, PS, and HRW within the same subfigure at *p* < 0.05.

The effects of the simulated condensation humidification treatment on the photosynthetic physiological parameters of the experimental plants are illustrated in Figure 9a–f. Following the 8 h condensation humidification, both the net photosynthetic rate (Pn) and transpiration rate (Tr) of *Pinus tabuliformis* and *Platycladus orientalis* exhibited an upward trend compared to their pre-experiment (Initial) levels. Specifically, the Pn of *Pinus tabuliformis* under the HRW treatment (Figure 9a) showed the highest increase, rising from a pre-experiment value of 8.46 µmol CO_2_ m^−2^ s^−1^ to a post-experiment value of 9.76 µmol CO_2_ m^−2^ s^−1^, which was superior to those of the CK treatment (9.27 µmol CO_2_ m^−2^ s^−1^) and the PS treatment (9.21 µmol CO_2_ m^−2^ s^−1^). *Platycladus orientalis* under the HRW treatment (Figure 9d) exhibited a similar pattern, with a pronounced increase in Tr, rising from 1.72 mmol H_2_O m^−2^ s^−1^ before the experiment to 2.35 mmol H_2_O m^−2^ s^−1^ after the experiment.

The variation trends in water use efficiency (WUE) are depicted in Figure 9e,f. Prior to the experiment, the WUE of *Pinus tabuliformis* and *Platycladus orientalis* were 3.19 µmol CO_2_ mmol^−1^ H_2_O and 1.81 µmol CO_2_ mmol^−1^ H_2_O, respectively. Post-experiment, the WUE across all treatment groups decreased compared to their initial values. Notably, the HRW treatment exhibited the lowest WUE levels in both tree species, recording 2.67 µmol CO_2_ mmol^−1^ H_2_O for *Pinus tabuliformis* and 1.46 µmol CO_2_ mmol^−1^ H_2_O for *Platycladus orientalis*.

## 4. Discussion

### 4.1. Construction of Artificial Microhabitats and Enhancement of Condensation Capture Efficiency via Hydrogel Application

The habitat conditions in the rocky mountainous regions of northern China are exceptionally harsh, characterized by shallow soil layers and high gravel content. These factors lead to restricted precipitation infiltration, increased surface runoff, and extremely poor water retention capacity, chronically subjecting vegetation to a severe water-limited state [7,8]. Under the backdrop of global climate change and the frequent occurrence of multi-year drought events [1,6], shifts in precipitation regimes have further exacerbated regional seasonal droughts [2,3]. High-frequency non-rainfall water inputs (NRWIs), such as condensation, serve as critical water sources for sustaining plant survival in this region [16,28]. However, the retention capacity of natural soil for such trace amounts of water is extremely limited, rendering it highly susceptible to evaporative loss following sunrise.

This study showed that the HRW treatment increased the net mass gain of the whole potted plant system during the simulated humidification period. This increase should be interpreted as enhanced system-level retention of externally supplied condensation water rather than as water uptake by the hydrogel alone. Notably, although the hydrogel was already fully hydrated at the onset of the experiment, its water status after placement in the root zone was likely dynamic rather than static. Once the prehydrated hydrogel came into contact with the surrounding soil and root system, water-potential gradients could drive part of the pre-loaded water to be gradually redistributed toward the rhizosphere. This internal redistribution would not itself increase the total system mass, but it may reduce the local swelling degree of the hydrogel and create additional capacity for retaining newly deposited condensation water. In this process, the hydrogel functioned as a dynamic water-exchange medium in the plant–soil–hydrogel continuum, retaining newly supplied condensation water near the root zone while redistributing stored water toward the soil–root interface. This is primarily attributed to the excellent hydrophilic network and extremely high hygroscopic potential of the polymer hydrogel. Consistent with the atmospheric water harvesting (AWH) mechanisms demonstrated by Lei et al. [29], the hydrogel matrix efficiently captures ambient water vapor and condensation, converting them in situ into non-volatile liquid water. Accordingly, the net mass gains of the HRW systems reached 26.3 g for *Pinus tabuliformis* and 32.9 g for *Platycladus orientalis*, which were 1.52 and 1.54 times those of their respective PS treatments. Furthermore, as comprehensively reviewed by Agbna and Zaidi [30], this hydrophilic polymer network utilizes capillary action and osmotic pressure to firmly retain the captured moisture, acting as a dynamic reservoir in the rhizosphere that significantly enhances the plants’ resilience to water deficits. The critical ecological role of this localized water retention is further corroborated by Mudhanganyi et al. [31], who demonstrated that such hydrogel-amended rhizospheres significantly improve the survival and early growth of Pinus seedlings during severe dry seasons by providing a sustained moisture buffer against drought stress.

Furthermore, the mass gain curve of the PS treatment for *Platycladus orientalis* was more linear compared to that of *Pinus tabuliformis*. This likely reflects that the scale-like leaves of *Platycladus orientalis* possess a larger contact area than the needles of *Pinus tabuliformis*, enabling a more effective capture of fine atmospheric mist droplets. However, while these specific leaf structures grant a high capacity to capture trace moisture—a mechanism also supported by the findings of Qiu et al. [32]—the absolute volume of water acquired through this isolated foliar pathway remains limited. Additionally, the condensation capture in the PS treatment of *Pinus tabuliformis* (relying solely on needle water uptake) was primarily concentrated in the early stages of humidification. As the leaf water content gradually increased, its needle water uptake rate significantly decreased, causing the system mass gain to level off at 17.3 g in the later stages of the experiment. In contrast, the foliar water uptake rate of *Platycladus orientalis* remained relatively stable throughout the entire experimental period.

In addition to root water uptake, foliar water uptake (FWU) is considered a widespread and ecologically significant water acquisition strategy across the plant kingdom [14,33,34]. This study utilized deuterium-enriched water (δ^2^H) tracing technology to precisely track water transport pathways. The experimental results demonstrated that the foliar δ^2^H values of *Pinus tabuliformis* and *Platycladus orientalis* exhibited a substantial positive shift following humidification, providing direct physiological evidence that these two typical afforestation tree species possess a significant FWU capacity. This is highly consistent with phenomena previously observed in coniferous species, cloud forest species, and arid-zone plants [35,36,37,38]. Plant leaves can directly capture and absorb condensation water through stomata, cuticles, or specific epidermal appendages [39,40]. Crucially, the post-experiment stem δ^2^H values of *Pinus tabuliformis* and *Platycladus orientalis* also increased significantly compared to their initial baselines, becoming enriched by 22‰ and 31‰, respectively. This confirms that the water absorbed by the leaves is not merely utilized to alleviate local canopy water deficits but is also translocated from the leaves to the stems. In traditional water transport models, water is absorbed by the roots and transported upward; however, under conditions of drought stress and canopy wetting, FWU can drive a reverse flow of water into the plant xylem and even down to the root system [17,41,42].

Integrating the aforementioned mechanisms, the quantitative analysis derived from the isotopic linear mixing model in this study revealed that under the HRW treatment, the fractional contribution of water converted and absorbed via the hydrogel medium to the stem water reached approximately 11%. This suggests a strong synergistic linkage between the microenvironment engineered by the hydrogel and the foliar water uptake mechanism of the plants. This profound integration of environmental material innovation with the plants’ intrinsic drought-tolerance mechanisms more efficiently restores drought-induced hydraulic dysfunction, thereby substantially enhancing the system’s conversion efficiency and utilization potential for condensation water.

### 4.2. Regulation of Plant Physiological Status and Utilization Strategies via Moisture Improvement

The enhancement of water availability directly drives adaptive adjustments in plant physiological metabolism and survival strategies [4,5]. In this study, following the treatment, the leaf water potential (Ψleaf, Figure 6), stomatal conductance (Gs, Figure 7), and leaf water content (LWC, Figure 8) of *Pinus tabuliformis* and *Platycladus orientalis* in the HRW treatment all exhibited substantial recovery, significantly outperforming the other two treatments. This indicates that a condensation environment can effectively alleviate plant water deficits. Consequently, despite this foliar interception, the recovery of physiological parameters in the CK and PS groups—which relied solely on natural condensation—was severely limited, with no significant differences observed between them. Furthermore, combining the system condensation mass gain data (Figure 3) with the stem water contributions (Figure 5), it becomes evident that although the bare soil surface of the CK group physically adsorbed a portion of the condensation (resulting in a higher mass gain than the PS group), this surface moisture failed to translate into tangible physiological benefits for the plants. Furthermore, the stem water source composition profiles of both groups were highly similar. This further confirms that trace amounts of condensation water absorbed by the surface soil are not easily transported upward via the root system within a short timeframe. This is primarily because the trace condensation water captured by the shallow soil is restricted by an extremely shallow infiltration depth; following sunrise, with increasing radiation and temperature, it is highly susceptible to evaporative loss and cannot be accessed by the deeper effective root zone [16,43].

Studies by Gotsch et al. [44], Eller et al. [45], and Breshears et al. [46] have confirmed that canopy wetting and foliar water uptake (FWU) during drought periods can significantly improve plant turgor pressure and water potential, mitigate the risk of xylem embolism, and restore photosynthetic gas exchange capacity. In this experiment, relying solely on foliar water uptake (PS) or minimal soil condensation water (CK) was insufficient to completely reverse the water deficit. The core ecological value of the hydrogel material (HRW) lies not only in its highly efficient capture of condensation water at night but also in its ability to supply water to the rhizosphere during the day. Its specialized three-dimensional network structure effectively locks in free water and, conforming to the root water potential gradient, rapidly and continuously delivers targeted moisture to the plant root system, thereby facilitating the rapid restoration of the plants to a higher physiological metabolic level within a short period [47].

In terms of plant physiological recovery, this study found that following the uptake of water replenished by the hydrogel, both the stomatal conductance (Gs) and net photosynthetic rate (Pn) of *Pinus tabuliformis* and *Platycladus orientalis* in the HRW group exhibited substantial increases. This result not only corroborates the previously mentioned leading advantages of the HRW group regarding leaf water potential (Ψleaf) and stomatal conductance (Gs) but also further demonstrates that the hydrogel medium can effectively capture and convert condensation water, thereby providing a more stable water source for the plants. This trend of physiological recovery is highly consistent with the findings of Leonel et al. [48], who observed that hydrogels can significantly improve the photosynthetic gas exchange efficiency of successional tree species during drought periods. Concurrently, research conducted by Mudhanganyi et al. [31] on *Pinus patula* seedlings also confirmed that hydrogels can substantially enhance the drought survival rate and growth performance of the plants by ameliorating the rhizosphere moisture environment. Collectively, these studies corroborate the value of hydrogels in alleviating water stress and facilitating comprehensive plant recovery.

Upon receiving ample water replenishment via both the foliage and the hydrogel, the stomata of the plants in the HRW group opened widely to maximize gas exchange. During this rapid recovery phase, the physical process of transpirational water loss increased at a rate that temporarily exceeded the biochemical recovery of the photosynthetic rate, resulting in a transient decrease in instantaneous water use efficiency (WUE). Rather than a physiological disadvantage, this dynamic reflects profound physiological plasticity in managing stomatal and diffusional limitations, a trait highly characteristic of drought-adapted species recovering from water deficits [49]. This behavior also represents a strong “compensatory effect” triggered by rehydration, enabling plants to rapidly upregulate gas exchange and capitalize on ephemeral water pulses for expedited metabolic recovery [50]. This demonstrates that these plants can successfully transition from a stringent ‘conservative water-saving’ strategy to an opportunistic ‘high-consumption’ mode to maximize survival and growth in arid environments.

The mechanism by which *Pinus tabuliformis* and *Platycladus orientalis* absorb condensation water via foliar water uptake and conduct reverse translocation demonstrates their intrinsic physiological drought-tolerance potential in coping with water-limited conditions. However, under the extreme site conditions of rocky mountainous regions, relying solely on transient nocturnal natural condensation water is insufficient to reverse the long-term negative water balance of the plants. The introduction of polymer hydrogels characterized by high water retention capacity not only intercepts and enriches near-surface water vapor at the physical level but also synergistically amplifies the capacity of the plant system to absorb and transport non-rainfall water at the biological level. The establishment of this “hydrogel-condensation-plant” coupled system breaks through the traditional afforestation paradigm that relies solely on soil precipitation replenishment. Ultimately, these findings will provide a theoretical basis and technical support for ecological afforestation and the highly efficient utilization of water resources in difficult sites, such as rocky mountainous areas.

### 4.3. Limitations and Future Research Directions

While this study successfully demonstrates the short-term efficacy of the eco-friendly hydrogel in enhancing condensation water utilization, certain limitations regarding the material itself must be acknowledged. Theoretically, the unique core–shell structure of the hydrogel endows it with the capacity for continuous, repeated water absorption and desorption. However, whether this structural integrity and functional performance can be sustainably maintained when incorporated into complex natural soil environments requires further verification. The natural soil matrix is highly heterogeneous, and extended exposure to physical compaction, chemical ion interactions, and microbial degradation in the rhizosphere may potentially compromise the three-dimensional porous network of the hydrogel over time.

Therefore, future research should prioritize investigating the long-term structural stability and degradation dynamics of this material under field conditions. Extensive in situ monitoring is necessary to evaluate how the water retention and release cycles of the hydrogel evolve over extended periods in natural soil profiles. Furthermore, future studies should broadly explore the functional diversity of this hydrogel. Beyond solely capturing condensation water, its extended ecological applications, such as regulating soil carbon and nitrogen distribution and influencing their mineralization characteristics in degraded habitats, warrant in-depth investigation. Addressing these aspects will provide a comprehensive evaluation of the ecological sustainability and multifunctionality of the material for large-scale afforestation in arid regions.

## 5. Conclusions

Through deuterium-enriched water (δ^2^H) tracing combined with a simulated condensation experiment, this study elucidated the absorption and transport dynamics of condensation water by two typical afforestation tree species (*Pinus tabuliformis* and *Platycladus orientalis*) in the rocky mountainous regions of northern China under the influence of a hydrogel. Furthermore, it systematically quantified their fractional water contributions and revealed the underlying mechanisms of this material in engineering microhabitats and regulating plant physiological responses. The main conclusions are as follows:

1. The hydrogel significantly enhanced the capture and accumulation efficiency of condensation water. Following 8 h of humidification, the HRW group exhibited the highest system net mass gain, reaching 26.3 g for *Pinus tabuliformis* and 32.9 g for *Platycladus orientalis*. These values were significantly higher than those of the control group (22.4 g and 25.4 g) and the pot-sealed group (17.3 g and 21.3 g), achieving a substantial enrichment of rhizosphere moisture within a short timeframe.
2. Both experimental plant species possess a significant foliar water uptake capacity. After humidification, the foliar δ^2^H values of *Platycladus orientalis* and *Pinus tabuliformis* surged to 113.5‰ and 85.3‰, respectively, while their stem δ^2^H values became enriched by 31‰ and 22‰ compared to their initial state. When relying solely on foliar water uptake, the fractional contributions of downwardly translocated condensation water to the stem water of *Platycladus orientalis* and *Pinus tabuliformis* reached 18.9% and 14.8%, respectively.
3. The synergy between the hydrogel and foliar water uptake maximized the physiological recovery of the plants. The water converted and absorbed by the hydrogel provided a supplemental contribution of 11.2% and 10.9% to the stem water of *Platycladus orientalis* and *Pinus tabuliformis*, respectively. Facilitated by this replenishment, the leaf water potentials of *Pinus tabuliformis* and *Platycladus orientalis* increased to −0.15 MPa and −1.32 MPa, stomatal conductance rose to 3.25 mm·s^−1^ and 3.64 mm·s^−1^, and leaf water content reached 58.4% and 67.4%. All these parameters were significantly superior to those in the treatment groups without hydrogel application.
4. The highly efficient water supply from the hydrogel prompted a significant transition in the physiological status of the plants. Following the alleviation of water limitation, the net photosynthetic rates and transpiration rates of the plants in the hydrogel group increased significantly, accompanied by a corresponding decrease in water use efficiency. Consequently, the plants transitioned from a stomatally limited state under water stress to a significantly more active state of gas exchange and physiological metabolism.

## Figures and Tables

**Table 1:** Plant growth status.

	Height/m	Diameter at Breast Height/cm	Planting Pot Diameter/cm	Planting Pot Height/cm	Soil Type
*Pinus* *tabuliformis*	1.5	2.1	33.5	33.3	Leached brown soil
*Platycladu* *orientalis*	1.5	1.8			

## Data Availability

The original contributions presented in this study are included in the article. Further inquiries can be directed to the corresponding authors.
